# Supplementary material for: A set of multi-entry identification keys to African frugivorous flies (Diptera, Tephritidae)
Source: Zookeys. 2014 Jul 24;(428):97–108. doi: 10.3897/zookeys.428.7366 (PMC4143993; doi:10.3897/zookeys.428.7366)
Supplement: Supplementary material 6 — Key to Ceratitis [file zookeys-428-097-s006.zip › SF6_ZooKeys_key to Ceratitis/key/SF6_ZooKeys_key to Ceratitis/Media/Html/Ceratitis cosyra .htm]

Ceratitis (Ceratalaspis) cosyra (Walker)


***Ceratitis*** **(*Ceratalaspis*)
*cosyra* (Walker)**

*Trypeta cosyra* Walker, 1849: 1042.

Body
length: 4.43 (3.35 - 5.40) mm; wing length: 4.17 (3.40-5.20) mm.

Male

Head.
Antenna yellow-orange. Third antennal segment twice as long as second segment.
Arista with short hairs over entire length. Frons with short scattered hairs
which are distinctly darker than or same colour as frons; more flattened, not
distinctly convex, in lateral view slightly projecting forwards at antennal
implant. Lower eye margin with slightly darker marking. Chaetotaxy normal for
subgenus, bristles dark reddish to black.

Thorax.
Postpronotum white, with black spot. Ground colour of mesonotum pale with
orange tinge; mesonotal pattern variable especially spots at mesal end of
suture and prescutellar spots variable in size and colouration, anterior
supra-alar spots usually continuous. Chaetotaxy normal for subgenus. Scapular
setae pale. One anepisternal bristle. Scutellum white basally, otherwise yellow
with three black separate markings apically; basally usually with two separate
dark spots, sometimes spots not outspoken, and only present as slightly brown
patches. Subscutellum pale with three dark separate spots except along dorsal
margin where touching.

Legs
yellow; setation typical for subgenus, mainly pale especially on femora.
Posterior and posterodorsal rows on fore femur pale. Ventral spines on fore
femur yellowish or black.

Wing
bands with markings extensively yellow; banding sometimes faint. Banding,
marginal band continuous; cubital band free; medial band absent; crossvein r-m
before middle of discal cell. Crossvein dm-cu position variable.

Abdomen.
Pale yellow or more brownish. Setation and banding typical for subgenus.

Female

As
male except for the following characters: Oviscape shorter than abdominal terga
3-6 combined.

(Description
after De Meyer, 1998)

��
